# Bridging High-Resolution Environmental Sensor Observations and Process-State Prediction: A Distribution-Shift-Robust Time–Frequency Transformer (FT-Crossformer)

**DOI:** 10.3390/s26165123

**Published:** 2026-08-13

**Authors:** Yiran Guan, Zhaoxu Yu, Hui Guo

**Affiliations:** School of Information Engineering, Ningxia University, Yinchuan 750021, China

**Keywords:** environmental sensor data, data assimilation, non-stationary sensor streams, bias correction, observation operator, multi-sensor fusion, multivariate time-series forecasting, Crossformer, distribution shift, frequency-domain analysis, wastewater treatment process, chlorophyll forecasting, estuarine monitoring

## Abstract

High-resolution online sensors are now common in environmental process systems, yet turning their non-stationary, heterogeneous observation streams into reliable predictions of the underlying process state remains difficult. The statistical distribution of a sensor stream changes over time, the measured variables do not coincide with the state variables of interest, and repeatedly running a mechanistic process model for forward prediction is computationally costly. We present FT-Crossformer, a time–frequency Transformer that acts as a data-driven surrogate between multi-sensor observations and multivariate process-state prediction. To handle distribution shift in the sensor streams, a time-domain distribution-transformation module, together with an inverse-mapping module, performs an affine bias correction that removes per-window non-stationary statistics at the input and restores them at the output, so the gap between training and test distributions is reduced without discarding non-stationary information. We show that this affine correction, including its learnable per-variable scale and shift, acts in the frequency domain on every non-zero frequency component as one common scaling factor that does not depend on the frequency index, so it cannot change the relative magnitudes among the components. A frequency-stability measurement module and a frequency-weighting module therefore re-weight the spectral components of the observation signal so that the stable, task-relevant ones contribute more to the reconstructed signal. The cross-dimension attention of the Crossformer backbone serves as a multi-sensor fusion mechanism that models the dependencies among the measured variables. We validate the method on public benchmark datasets from different domains as a check of generality and, most relevantly, for environmental modeling on two real cases: a wastewater nitrogen-and-phosphorus-removal process and chlorophyll forecasting from an in situ estuary sensor mooring in San Francisco Bay. On the estuary chlorophyll data, which carries a strong train-to-test distribution shift, the full FT-Crossformer demonstrates superior accuracy among the evaluated models at the next-day nowcasting horizon, and an ablation shows that both the time-domain trans- formation and the frequency-domain weighting contribute to this accuracy. FT-Crossformer produces forward predictions from distribution-shifted sensor data with a single fixed-cost forward pass in place of a repeated mechanistic solve, which makes it a practical building block for sensor-data integration and assimilation in environmental process modeling.

## 1. Introduction

The spread of high-resolution online sensing in environmental engineering, such as dissolved-oxygen, ammonia, nitrate, phosphate and mixed-liquor-suspended-solids probes in wastewater treatment, as well as in aquatic ecological monitoring with in situ chlorophyll-a optical sensors, has produced a large stream of observational data, and at the same time it has exposed two well-known obstacles to turning that data into actionable predictions. Chlorophyll-a time series, a core biomarker for algal bloom and eutrophication warning, presents obvious multi-period oscillations driven by diurnal light cycles and seasonal hydrological variation, further complicating reliable forecasting. The first obstacle is computational. A mechanistic environmental process model, for example, an activated sludge model [1], is expensive to solve repeatedly for forward prediction, which motivates a fast data-driven surrogate that maps recent observations directly to future states. For aquatic ecology, complex biogeochemical mechanistic models similarly require heavy computation for high-frequency chlorophyll simulation. The second obstacle is that the raw sensor measurements are non-stationary and do not map directly onto the process state variables of interest: their statistical distribution changes over time as instruments age, zero points drift and the measurement environment varies, which degrades the accuracy and the generalization of a standard forecasting model. For chlorophyll monitoring, hydrodynamic shifts, seasonal temperature turnover and seasonal phytoplankton succession incur severe distribution shift between training sensor records and real prediction periods, causing prominent performance decay in conventional time-domain prediction models. Existing time–frequency chlorophyll prediction tools mostly focus on periodic decomposition rather than resisting distribution drift, lacking cross-scenario generalization capacity [2]. We address both obstacles with a single time–frequency Transformer that learns an operator from distribution-shifted multi-sensor observations to multivariate process-state predictions, specifically accommodating high-frequency chlorophyll and conventional water quality sensor signals.

We use a multivariate time series as the data structure for a sensor stream. A multivariate time series is a matrix $X\in R^{T\times D}$ whose entry $x_{t,d}$ is the value of the d-th sensor at the t-th sampling instant, where T is the number of historical time steps and D is the number of sensors. The forecasting task is to predict the matrix $Y\in R^{\tau \times D}$ of the next τ steps. Such data carries a temporal pattern along the time axis and a nonlinear dependency among the variables along the sensor axis, and a forecasting model must capture both. Crossformer [3], a Transformer variant, models both dependencies through a dimension-segment embedding and a two-stage attention mechanism, and uses the information of correlated sensors to improve a prediction. Crossformer does not, however, account for a change in the underlying data-generating process, that is, for distribution shift. It assumes that the training data and the test data follow one distribution, whereas in an environmental process the two are split by time, so the distribution of the measured data changes and the predictive performance of Crossformer falls. Shao et al. [4] report the same effect: on a dataset whose distribution changes, a Transformer-based model tends to overfit and its performance drops markedly.

A second consideration concerns the domain of analysis. A multivariate time-series model usually extracts features and is built in the time domain, but the periodic, trend and fluctuation components of a series are often clearer in the frequency domain, because a series can be written as a superposition of frequency components [5,6]. Some periodic and trend structures of a series are represented more directly by a small number of frequency components than by the raw time-domain samples, so a model that operates in the time domain alone has no explicit access to that representation.

The contributions of this work are as follows. We propose FT-Crossformer, a time–frequency joint-modeling method built on the Crossformer backbone that addresses distribution shift in environmental sensor streams. We add a distribution-transformation module and an inverse-mapping module that form a symmetric affine pair, which removes the per-window non-stationary statistics of the input and restores them at the output, so the training–test distribution gap is reduced without losing non-stationary information. We show that this affine correction, with its learnable per-variable scale and shift, scales every non-zero frequency component by one factor that is the same for all frequency indices, leaving the relative magnitudes among the components unchanged, and we add a frequency-stability measurement module and a frequency-weighting module that re-weight the spectral components so that the stable, task-relevant ones contribute more to the reconstructed signal. We validate the method on public benchmarks and on two real environmental cases, a wastewater nitrogen-and-phosphorus-removal process and chlorophyll forecasting from an estuary sensor mooring, and we discuss the relation of the model to mechanistic models and to data assimilation. On the estuary chlorophyll data, the full model appears to be the most accurate at the next-day nowcasting horizon, and an ablation shows that both proposed components contribute to this accuracy.

## 2. Related Work

Time-series forecasting divides into classical statistical methods and modern deep-learning methods. The statistical approach to multivariate forecasting includes the autoregressive moving-average model (ARMA) [7] and the autoregressive integrated moving-average model (ARIMA) [8]. When the data is multivariate, non-stationary and strongly nonlinear, these methods lead to low accuracy at a high computational cost. Deep learning has advanced the field through its representation power and its flexible network design; the common models are the temporal convolutional network (TCN) [9], the long short-term memory network (LSTM) [10] and the Transformer [11].

Among the deep-learning models, the Transformer captures a long-range dependency through self-attention, which computes an attention weight between any two positions of a sequence. Informer [12] introduces ProbSparse self-attention and a distilling operation to reduce the quadratic complexity of the Transformer. Autoformer [13] uses a decomposition architecture that separates a series into a trend term and a seasonal term and an auto-correlation mechanism designed for periodic structure, whose benefit is smaller when the series is not periodic. FEDformer [14] applies attention in the frequency domain through Fourier and wavelet transforms and selects a fixed-size random subset of the spectrum to obtain linear complexity. iTransformer [15] replaces the self-attention with a multilayer perceptron and applies the model to the feature dimension rather than the time dimension.

These Transformer-based models perform well in several settings, but their accuracy still reduces for multivariate data with distribution shift. Rabanser et al. [16] compare unsupervised shift-detection methods and propose a two-sample hypothesis test; Lipton et al. [17] propose a hypothesis test for black-box shift estimation, and Kulinski et al. [18] propose an expected-conditional-distance statistic that locates the shifted features. These methods detect and locate a shift, whereas the present method corrects it. Domain adaptation [19,20,21] reduces the gap between a source domain and a target domain, and domain generalization [22,23,24] trains on the source domain and aims for good performance on the target domain; both seek to shrink the gap between the original distribution and the target distribution. In an environmental process, however, the distribution shift is continuous and dynamic in time, so the boundary of a domain is hard to define, which limits these methods. Recent work in the environmental and Earth-system sciences frames a learned model either as a surrogate for an expensive forward operator or as a learned observation operator that complements assimilation [25,26,27]; the data-driven modeling of wastewater treatment plants [28] and machine-learning retrieval of ocean variables [29,30] are concrete instances. Our method sits in this line of work as a bias-corrected surrogate for process-state prediction.

## 3. Methodology

### 3.1. Crossformer Backbone

Crossformer captures the cross-time and cross-dimension dependencies of a multivariate time series through a dimension-segment embedding and a two-stage attention layer.

#### 3.1.1. Dimension-Segment Embedding

Let the historical multivariate series be $X_{1:T}\in R^{T\times D}$ and let the target be the next τ steps $X_{T+1:T+\tau }$, where T is the number of historical steps and D is the number of variables. Rather than embedding the several variables at one timestamp, the dimension-segment embedding treats a run of consecutive values of a single variable as one embedding vector, so that the information of neighboring instants is preserved. Each variable is first cut into segments of length $L_{seg}$. We assume $L_{seg}$ divides T and partition the time index {1,…,T} into the consecutive blocks indexed by i with $1\leq i\leq T/L_{seg}$. The i-th segment of variable d, where 1≤d≤D, is the ordered tuple of the values on the i-th block,

(1) $$x_{i,d}^{\left(s\right)}=(x_{t,d}{)}_{\left(i-1\right)L_{seg}<t\leq i\;L_{seg}}\in R^{L_{seg}},$$

where t is the time step, the entries of $x_{i,d}^{\left(s\right)}$ are listed in increasing order of t, so $x_{i,d}^{\left(s\right)}$ is a vector in $R^{L_{seg}}$ and not an unordered set, and the collection $\{x_{i,d}^{\left(s\right)}{\}}_{i,d}$ is a reindexing of the matrix $X_{1:T}$. Each segment is embedded by a linear projection with a position embedding,

(2) $$h_{i,d}=E\;x_{i,d}^{\left(s\right)}+E_{i,d}^{\left(pos\right)},$$

where E is a learnable projection matrix and $E_{i,d}^{\left(pos\right)}$ is the learnable position embedding of segment i in variable d. The result is a two-dimensional array $H=\{h_{i,d}\}$ whose two axes correspond to time and dimension, and which is the input of the two-stage attention layer.

#### 3.1.2. Two-Stage Attention

The two-stage attention models the cross-time and the cross-dimension dependency in turn. Write the input array as Z; let $Z_{i,:}$ be the vector of all dimensions at time step i and $Z_{:,d}$ the vector of all time steps in dimension d. The cross-time stage applies multi-head self-attention within each dimension,

(3) $$\begin{array}{c} {\hat{Z}}_{:,d}=Z_{:,d}+\mathrm{MSA}\left(Z_{:,d},Z_{:,d},Z_{:,d}\right), \end{array}$$

(4) $$\begin{array}{c} Z_{:,d}^{time}={\hat{Z}}_{:,d}+\mathrm{MLP}\left(LayerNorm({\hat{Z}}_{:,d})\right), \end{array}$$

where LayerNorm is layer normalization, MSA is multi-head self-attention and MLP is a feed-forward network; the dependency among the segments of one dimension is captured in $Z^{time}$. To avoid the high cost of attention over the full array, the cross-dimension stage uses a small fixed number c of learnable router vectors at each time step i,

(5) $$\begin{array}{c} B_{i,:}=MSA\left(R_{i,:},Z_{i,:}^{time},Z_{i,:}^{time}\right), \end{array}$$

(6) $$\begin{array}{c} {\bar{Z}}_{i,:}=MSA\left(Z_{i,:}^{time},B_{i,:},B_{i,:}\right), \end{array}$$

(7) $$\begin{array}{c} {\tilde{Z}}_{i,:}=Z_{i,:}^{time}+{\bar{Z}}_{i,:}, \end{array}$$

(8) $$\begin{array}{c} Z_{i,:}^{dim}={\tilde{Z}}_{i,:}+MLP\left(LayerNorm({\tilde{Z}}_{i,:})\right), \end{array}$$

where $R_{i,:}$ is the array of c router vectors at time step i, $B_{i,:}$ is the information the routers gather from all dimensions, ${\bar{Z}}_{i,:}$ is the router output distributed back to the dimensions, ${\tilde{Z}}_{i,:}$ is the residual output and $Z_{i,:}^{dim}$ is the output of the feed-forward network. The cross-time and the cross-dimension dependency are then both held in $Z^{dim}$.

### 3.2. Time-Domain Transformation

Distribution shift is a change in the underlying data-generating process, often caused by an observed or unobserved context [4]; the segment embedding of Crossformer preserves more of that local context than a per-timestamp embedding, which makes the backbone a suitable base. The cause of the shift is that the statistical properties of the data change over time. Removing the non-stationary information at the input reduces the gap, but the non-stationary information is needed for the prediction to keep its physical meaning, so it cannot simply be discarded.

To address this, we incorporate an affine transformation into the Crossformer model, comprising a transformation layer and its inverse, arranged symmetrically. The transformation layer acts on the input and re-centers the distribution at its mean, temporarily removing the non-stationary information. The inverse layer acts on the prediction and restores it to the original scale, so the gap between the training and the test distribution is reduced while the original distribution is preserved. For a batch of N multivariate input series $X^{\left(n\right)}$, each with T historical steps and D variables and used to predict the next τ steps, the transformation module first computes, for each instance, the per-window mean and standard deviation,

(9) $$\begin{array}{c} \mu_{d}^{\left(n\right)}=\frac{1}{T}\sum_{t=1}^{T}x_{t,d}^{\left(n\right)}, \end{array}$$

(10) $$\begin{array}{c} \sigma_{d}^{\left(n\right)}=\sqrt{\frac{1}{T}\sum_{t=1}^{T}{\left(x_{t,d}^{\left(n\right)}-\mu_{d}^{\left(n\right)}\right)}^{2}}, \end{array}$$

where $x_{t,d}^{\left(n\right)}$ is the value of variable d at time step t in the n-th series, and where the training distribution $P_{train}(X)$ and the test distribution $P_{test}(X)$ differ. The module then normalizes the input,

(11) $${\hat{x}}_{t,d}^{\left(n\right)}=\gamma_{d}⊙\frac{x_{t,d}^{\left(n\right)}-\mu_{d}^{\left(n\right)}}{\sqrt{{\left(\sigma_{d}^{\left(n\right)}\right)}^{2}+\epsilon }}+\beta_{d},$$

where $\gamma_{d}$ and $\beta_{d}$ are learnable affine scale and shift parameters, ⊙ is the Hadamard product and ϵ is a small constant for numerical stability. The scale $\gamma_{d}$ is initialized to 1 and the inverse layer below divides by this, so we keep $\gamma_{d}$ bounded away from zero through this initialization and through weight decay during training. The statistics $\mu_{d}^{\left(n\right)}$, $\sigma_{d}^{\left(n\right)}$, $\gamma_{d}$ and $\beta_{d}$ are stored for the inverse layer.

The transformed series $\hat{X}$ is the input of the encoder–decoder. In the encoder, every layer except the first merges two adjacent vectors in time to obtain a coarser representation and then applies the two-stage attention,

(12) $$\begin{array}{c} {\hat{H}}_{i,d}^{l}=W^{merge}\left[\;H_{2i-1,d}^{l-1}\;∥\;H_{2i,d}^{l-1}\;\right], \end{array}$$

(13) $$\begin{array}{c} H^{l}=TSA\left({\hat{H}}^{l}\right), \end{array}$$

where H is a two-dimensional array, $W^{merge}$ is a learnable matrix, ∥ denotes concatenation, ${\hat{H}}^{l}$ is the merged array, TSA is the two-stage attention and $H^{l}$ is the output of encoder layer l. If the encoder has M layers, the decoder uses M+1 layers,

(14) $$\begin{array}{c} {\tilde{H}}^{l}=TSA\left(H_{l-1}^{\left(dec\right)}+E_{l}^{\left(dec\right)}\right), \end{array}$$

(15) $$\begin{array}{c} H_{l}^{\left(dec\right)}={\tilde{H}}^{l}+cross\left({\tilde{H}}^{l},H^{l}\right), \end{array}$$

(16) $$\begin{array}{c} Y^{l}=H_{l}^{\left(dec\right)}+MLP\left(LayerNorm(H_{l}^{\left(dec\right)})\right), \end{array}$$

where $E_{l}^{\left(dec\right)}$ is the learnable position embedding of the decoder, ${\tilde{H}}^{l}$ is the two-stage attention output, $H_{l}^{\left(dec\right)}$ is the output after connecting the encoder and decoder, and $Y^{l}$ is the output after the residual connection and the feed-forward network. A linear projection of each layer produces that layer’s prediction, and the layer predictions are summed to give $\hat{Y}$.

**Figure 1:**
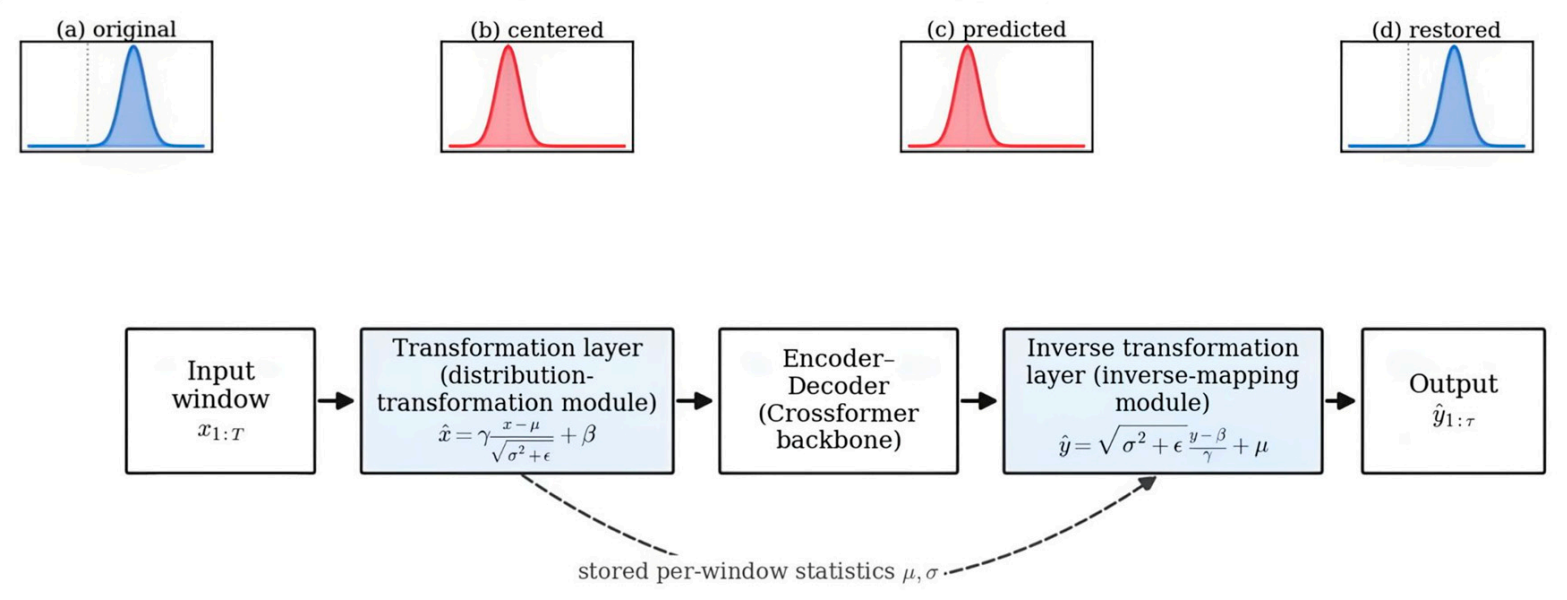
Time-domain transformation. The distribution-transformation module removes the per-window non-stationary statistics (mean and standard deviation) at the input, and the inverse-mapping module restores them at the output. The insets (**a**–**d**) show the per-window distribution of the original input, of the transformed input, of the prediction in the transformed space, and of the output after inverse mapping.

To restore the physical meaning, the prediction $\hat{Y}$ is passed to the inverse layer, which uses the stored statistics to invert the transformation,

(17) $$X_{k}^{\left(n\right)}=\sum_{t=0}^{T-1}x_{t}^{\left(n\right)}\;e^{-\;i\;2\pi kt/T},\;k=0,1,\ldots ,K-1,$$

which yields the output Y on the original scale. Figure 1 shows the two layers together with the per-window distribution at each stage of the pipeline.

### 3.3. Frequency-Domain Weighting

**Figure 2:**
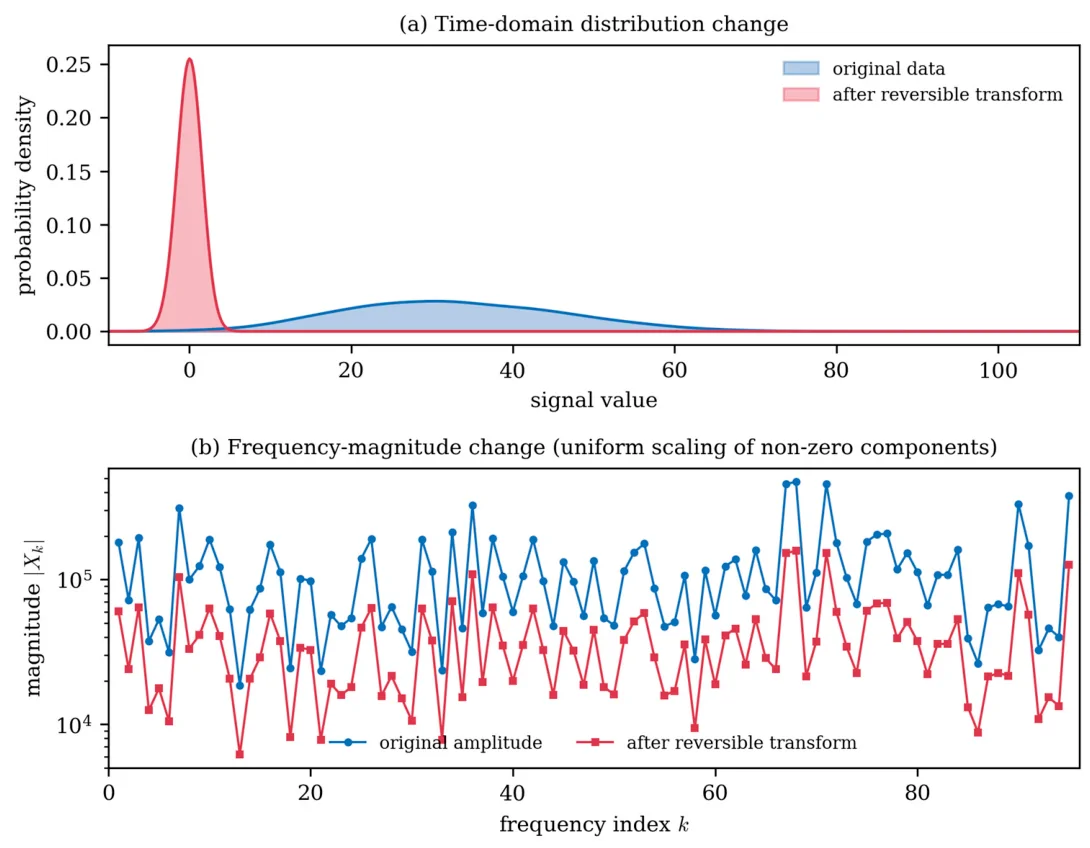
Effect of the time-domain transformation in the two domains. (**a**) In the time domain, it maps the original signal distribution to a zero-centered distribution. (**b**) In the frequency domain, it scales every non-zero component by a single common factor, so the relative magnitudes of the components are unchanged; this is the blurring of the task-relevant frequency components that the frequency-domain weighting is designed to undo.

The time-domain transformation reduces the distribution shift in the time domain, but it creates a new problem in the frequency domain. As shown below, the transformation maps the original data to a more compact, mean-centered distribution in the time domain, yet in the frequency domain it multiplies every non-zero frequency component by one factor that is the same for all frequency indices, so the relative magnitudes among the components do not change. The transformation thus gives no extra weight to the components that carry the task-relevant features, and the result remains sensitive to noise in the other components. To tackle this challenge, we propose a novel frequency-domain weighting mechanism, which integrates a frequency-stability measurement module for quantifying component stability and an adaptive frequency-weighting module for dynamic component re-weighting. Figure 2 contrasts the effect of the transformation in the two domains and makes this point precise.

Given a dataset of N series, consider one scalar channel of the n-th sample over the window of length T. Its discrete Fourier transform is

(18) $$X_{k}^{\left(n\right)}=\sum_{t=0}^{T-1}x_{t}^{\left(n\right)}\;e^{-\;i\;2\pi kt/T},\;k=0,1,\ldots ,K-1,$$

where each $X_{k}^{\left(n\right)}$ is a complex scalar. A length-T DFT of a real signal is conjugate-symmetric, so the non-negative frequencies determine the spectrum; we keep these and set K=⌊T/2⌋+1, so the frequency index runs k=0,1,…,K−1. The quantity $\left|X_{k}^{\left(n\right)}\right|$ is the modulus of the complex coefficient $X_{k}^{\left(n\right)}$, that is, the magnitude of frequency component k. To quantify stability we use a measure based on the coefficient of variation [31], the ratio of the standard deviation to the mean of a component’s magnitude taken over the N training samples,

(19) $${CV}_{k}=\frac{\sigma_{\left|X_{k}\right|}}{\mu_{\left|X_{k}\right|}},\;\mu_{\left|X_{k}\right|}=\frac{1}{N}\sum_{n=1}^{N}|X_{k}^{\left(n\right)}|,$$

where $\mu_{\left|X_{k}\right|}$ and $\sigma_{\left|X_{k}\right|}$ are the mean and the standard deviation of the magnitude of component k across the N samples. A component with a smaller coefficient of variation has a magnitude that is more nearly constant across samples, and we call it more stable. Fixing a threshold $c_{0}$, the stable set is

(20) $$S=\{k:{CV}_{k}\leq c_{0}\},$$

the set of frequency indices whose magnitude varies little across the training samples. The measure ${CV}_{k}$ and the set S enter the weighting module below through the initialization of its weights.

We now show that the full affine transformation of Equation (11), including its learnable scale and shift, acts on each non-zero frequency as a single scaling factor that does not depend on the frequency index. Fix one variable d and one window, and abbreviate $\mu =\mu_{d}$, $\sigma =\sigma_{d}$, $\gamma =\gamma_{d}$, $\beta =\beta_{d}$. The transformed series is

(21) $${\hat{x}}_{t}=\gamma \;\frac{x_{t}-\mu }{\sqrt{\sigma^{2}+\epsilon }}+\beta .$$

Take the DFT and use its linearity,

(22) $${\hat{X}}_{k}=\frac{\gamma }{\sqrt{\sigma^{2}+\epsilon }}\sum_{t=0}^{T-1}x_{t}\;e^{-\;i\;2\pi kt/T}+\left(\beta -\frac{\gamma \mu }{\sqrt{\sigma^{2}+\epsilon }}\right)\sum_{t=0}^{T-1}e^{-\;i\;2\pi kt/T}.$$

The first sum is $X_{k}$ by definition. The second sum is a geometric series in $e^{-i\;2\pi k/T}$, which equals T at k=0 and 0 for every other k in 0,…,T−1; write this as $T\;\delta_{k}$, where $\delta_{k}$ is 1 at k=0 and 0 otherwise. Hence,

(23) $${\hat{X}}_{k}=\frac{\gamma }{\sqrt{\sigma^{2}+\epsilon }}\;X_{k}+\left(\beta -\frac{\gamma \mu }{\sqrt{\sigma^{2}+\epsilon }}\right)\;T\;\delta_{k}.$$

For a non-zero frequency k≠0 the constant term drops, so

(24) $${\hat{X}}_{k}=\frac{\gamma }{\sqrt{\sigma^{2}+\epsilon }}\;X_{k},\;\;|{\hat{X}}_{k}|=\frac{\gamma }{\sqrt{\sigma^{2}+\epsilon }}\;|X_{k}|,$$

where $\left|{\hat{X}}_{k}\right|$ is the magnitude of component k after the transformation. The learnable shift β and the mean μ contribute only at k=0, that is, only to the DC bin. For every k≠0, the affine transformation multiplies the coefficient by the single factor $\gamma /\sqrt{\sigma^{2}+\epsilon }$, which is the same for all non-zero frequency indices and depends only on the variable d through $\gamma_{d}$ and $\sigma_{d}$. The transformation therefore leaves the ratios $\left|{\hat{X}}_{k}|/|{\hat{X}}_{k^{\prime }}\right|$ among non-zero frequencies unchanged, so it cannot by itself separate the task-relevant components, which motivates the weighting below.

In the frequency-weighting module, the series is first smoothed by a first-order difference and then transformed; the spectrum is split into its real and imaginary parts.

(25) $$\begin{array}{c} \Delta x_{t}=x_{t}-x_{t-1}, \end{array}$$

(26) $$\begin{array}{c} F(\Delta x{)}_{k}=R_{k}+i\;I_{k}, \end{array}$$

(27) $$\begin{array}{c} (R,I)=\left(R\;F(\Delta x),\;I\;F(\Delta x)\right), \end{array}$$

where Δ is the difference operator, F(Δx) is the DFT of the differenced series on the same K=⌊T/2⌋+1 non-negative frequencies, and R and I are the real-part and imaginary-part vectors indexed by k=0,…,K−1. A learnable linear projection is applied to each part and combined with the original part by a Hadamard product, which re-weights the components,

(28) $$\begin{array}{c} \tilde{R}=R⊙\left(W_{R}\;R+b_{R}\right), \end{array}$$

(29) $$\begin{array}{c} \tilde{I}=I⊙\left(W_{I}\;I+b_{I}\right), \end{array}$$

where $W_{R}$ and $W_{I}$ are the weight matrices of the real and the imaginary part and $b_{R}$ and $b_{I}$ are bias vectors. We separately project the real and imaginary parts—corresponding to the cosine and sine components—at a merely linear computational cost, thereby enabling their independent re-weighting. The stability measure couples to this re-weighting through the initialization: we set the bias so that the initial per-index gain $W_{R}R+b_{R}$ (and likewise for I) starts near 1 on the stable set S and near 0 off it, that is, the initialization passes the components in S and damps the rest:

(30) $$(b_{R}{)}_{k}=(b_{I}{)}_{k}=\left\{\begin{array}{ll} 1, & k\in S,\\ 0, & k\notin S, \end{array}\right.\;\;W_{R},W_{I}\;\mathrm{initialized}\;\mathrm{to}\;0.$$

With the aforementioned initialization, while stable components are held constant initially, the sequential adaptive iteration of parameters $W_{R},W_{I},b_{R},b_{I}$ and S during training enables the weighted reinforcement of the stable or task-relevant components and the concomitant attenuation of non-stable ones, through which the final weights are learned. The re-weighted parts are returned to the time domain by the inverse transform that matches the one-sided spectrum above,

(31) $${\tilde{x}}_{t}=\frac{1}{T}\sum_{k=0}^{T-1}\left({\tilde{R}}_{k}+i\;{\tilde{I}}_{k}\right)e^{\;i\;2\pi kt/T},$$

where for k>⌊T/2⌋ the coefficients ${\tilde{R}}_{k}+i\;{\tilde{I}}_{k}$ are filled in by conjugate symmetry from the non-negative frequencies, so the sum runs over all T bins and $\tilde{x}$ is real and has the same length as x.

### 3.4. Full Model

**Figure 3:**
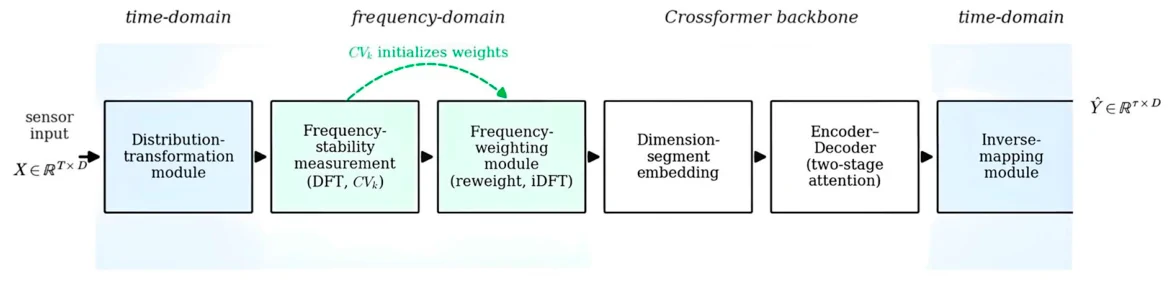
FT-Crossformer architecture. A time-domain distribution-transformation and inverse-mapping pair wraps the Crossformer backbone; between them, a frequency-stability measurement module and a frequency-weighting module re-weight the stable spectral components, after which dimension-segment embedding and two-stage attention produce the prediction.

Figure 3 shows how these modules are assembled. FT-Crossformer combines the dimension-segment embedding, the two-stage attention, the distribution-transformation module, the inverse-mapping module, the frequency-stability measurement module, the frequency-weighting module and the encoder–decoder. The historical data first enters the distribution-transformation module, which re-centers the input distribution at its mean and temporarily removes the non-stationary statistics. The frequency-stability measurement module then transforms the data to the frequency domain by the DFT, measures the coefficient of variation in each component across the training samples and forms the stable set S; this set initializes the weights of the frequency-weighting module, which re-weights the spectral representation by the learnable linear projection of Equations (28)–(30) and returns to the time domain by the inverse DFT. The dimension-segment embedding splits the series into segments, and the embedded output enters the multilayer encoder–decoder, where each encoder layer applies the two-stage attention to capture the cross-time and the cross-dimension dependency and the decoder forms a prediction layer by layer. The inverse-mapping module then restores the distribution and produces the final prediction. The distribution shift is corrected in the time domain, and in the frequency domain the re-weighting starts from the stable components and is then learned, which together improve the accuracy and the generalization of the model.

## 4. Experiments

We first test the method on public benchmarks from different domains to establish that it forecasts under distribution shift beyond a single application. We then run an ablation, and we report two real environmental cases: chlorophyll forecasting from an estuary sensor mooring and a wastewater nitrogen-and-phosphorus-removal process. A final subsection relates the model to mechanistic models and to data assimilation, and discusses inference cost.

### 4.1. Public Benchmarks: A Check of Generality

We use five widely used public datasets that span energy, traffic, weather, public health and economics. The Electricity Transformer Temperature (ETT) dataset [12] contains seven factors of an electricity transformer, including load features and oil temperature, recorded hourly (ETTh1, ETTh2) and every fifteen minutes (ETTm1, ETTm2). The Influenza Like Illness (ILI) dataset [13] is the weekly ratio of influenza patients to all patients from the US Centers for Disease Control and Prevention between 2002 and 2021. Traffic [13] is the hourly road-occupancy rate from 862 sensors on San Francisco Bay Area freeways between January 2015 and December 2016. Weather [13] is 21 meteorological factors recorded every ten minutes in 2020 at the Max Planck Institute for Biogeochemistry, Bremen, Germany. Exchange Rate [13] is the daily exchange-rate panel of eight countries between 1990 and 2016, which carries a long-term trend and a random walk.

We compare FT-Crossformer with six typical models: iTransformer [15], DLinear [32], Crossformer [3], FEDformer [14], Autoformer [13] and Informer [12]. Each split is normalized with the mean and the standard deviation of the training set. All experiments were performed on a workstation running Ubuntu 20.04.1 LTS, equipped with an NVIDIA RTX A6000 96GB GPU and an Intel Xeon Gold 6326 @ 2.90GHz CPU. The implementation was built upon PyTorch 1.13.1 with CUDA 11.7 support. The proposed model adopts an enhanced Crossformer architecture consisting of three encoder layers and four decoder layers; the number of routers in the two-stage attention is ten; the hidden state has dimension 256; multi-head attention uses four heads; the historical window is 96. The segment length is six when the prediction length is below 168 and 24 otherwise; the batch size is 32, reduced to 8 on Traffic to fit memory. Training uses Adam, with the initial learning rate chosen from $\left\{5\times 10^{-3},10^{-3},5\times 10^{-4},10^{-4},5\times 10^{-5},10^{-5}\right\}$ by grid search and halved each epoch; the loss is the mean-squared error, dropout is 0.2, normalization is layer normalization, the activation is GELU and the attention weights use a softmax. Training runs for 20 epochs with early stopping after three epochs without an improvement in the validation loss. The implementation uses PyTorch and each result is the mean of three runs.

We report the mean absolute error (MAE) and the mean-squared error (MSE),

(32) $$\begin{array}{c} MAE=\frac{1}{n}\sum_{i=1}^{n}\left|y_{i}-{\hat{y}}_{i}\right|, \end{array}$$

(33) $$\begin{array}{c} MSE=\frac{1}{n}\sum_{i=1}^{n}{\left(y_{i}-{\hat{y}}_{i}\right)}^{2}, \end{array}$$

where n is the length of the test set, $y_{i}$ is the true value and ${\hat{y}}_{i}$ is the prediction; both are in [0,+∞), both equal zero at a perfect prediction, and a lower value is better.

**Figure 4:**
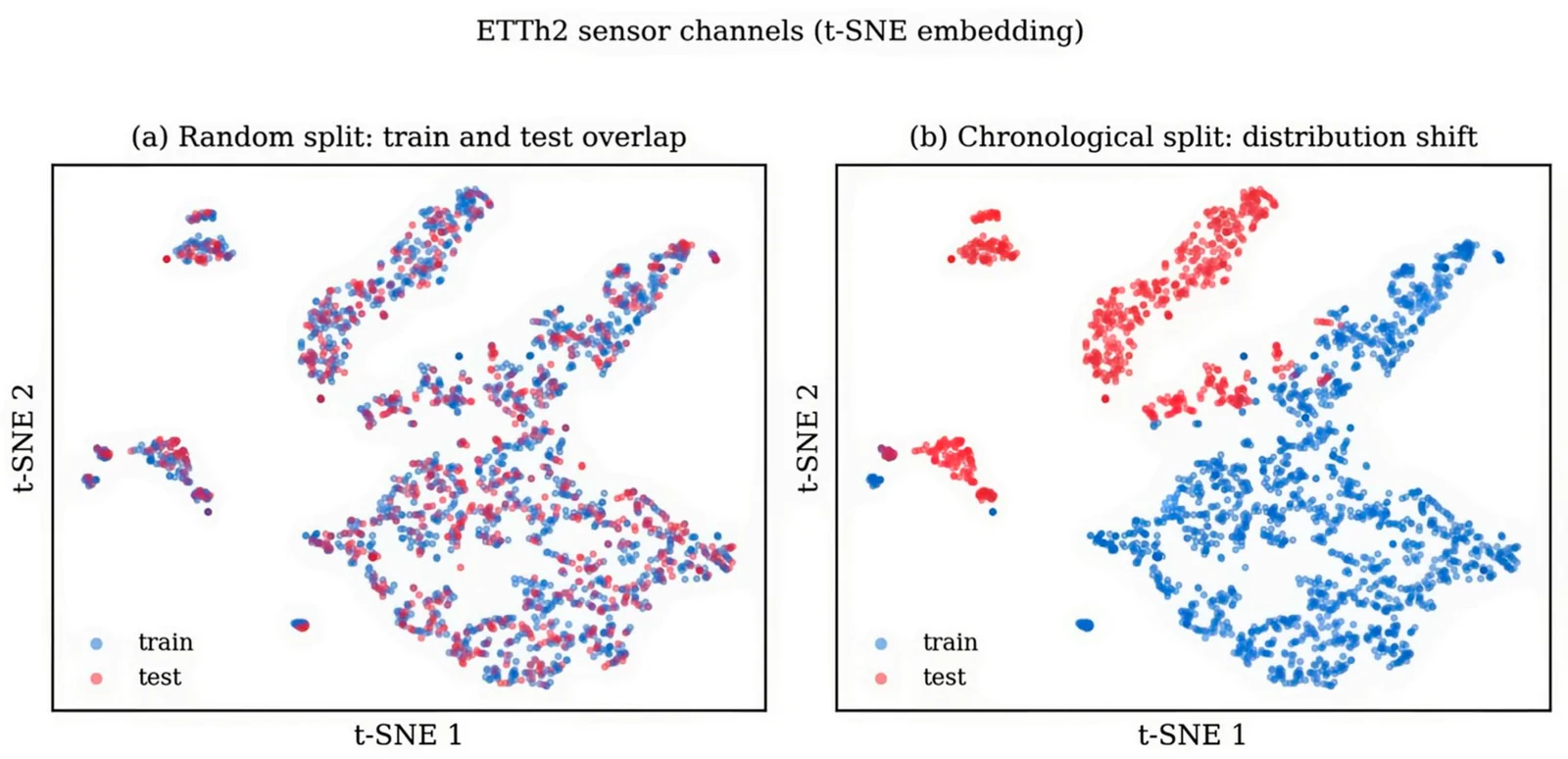
Distribution shift under a chronological split, illustrated on the ETTh2 benchmark by a t-SNE embedding of the sensor channels. (**a**) Under a random split, the training and test points overlap. (**b**) Under the chronological split used for forecasting, the test points occupy a distinct region of the embedding, i.e., the training and test distributions differ.

Table 1 reports the comparison over the datasets and the prediction lengths. FT-Crossformer obtains the best result on every prediction length of every dataset except two long-horizon cases on Traffic. At τ=168 and τ=336 on Traffic, iTransformer attains a lower MSE while FT-Crossformer still attains the lower MAE; at the other prediction lengths FT-Crossformer demonstrates superior accuracy on both metrics. Relative to the original Crossformer, the average MSE drops by about 26.0% on ETTh2 and by about 20.7% on Traffic, so the method improves over the backbone on both datasets. The gain on ETTh2 is consistent with the train/test distribution shift that the chronological split induces: Figure 4 embeds the ETTh2 sensor channels with t-SNE, and under a random split (panel a) the training and test points overlap, whereas under the chronological split used for forecasting (panel b) the test points occupy a region of the embedding distinct from the training points.

### 4.2. Ablation Study

We remove one component at a time to measure its contribution. “w/o FDW” removes the frequency-stability measurement module and the frequency-weighting module; “w/o DTIM” removes the distribution-transformation module and the inverse-mapping module; “w/o DSETA” removes the dimension-segment embedding and the two-stage attention, so the model embeds the several variables at a single time point and captures only the temporal dependency. Table 2 reports the result on ETTh2 and ILI. Removing FDW raises both MSE and MAE on every prediction length, more so at the long horizons, which shows that the frequency-weighting module contributes to the accuracy; the mechanism we attribute to it is the re-weighting of the spectral components from the stable set (Equations (28)–(30)), which is the design intent rather than a quantity the ablation isolates on its own. Removing DTIM or DSETA raises every metric markedly, which shows that both the dimension-segment embedding and the distribution transformation contribute; we read this as the segment embedding retaining the local context and the distribution transformation reducing the shift before prediction.

### 4.3. Chlorophyll Forecasting from Estuarine Sensor Moorings

**Figure 5:**
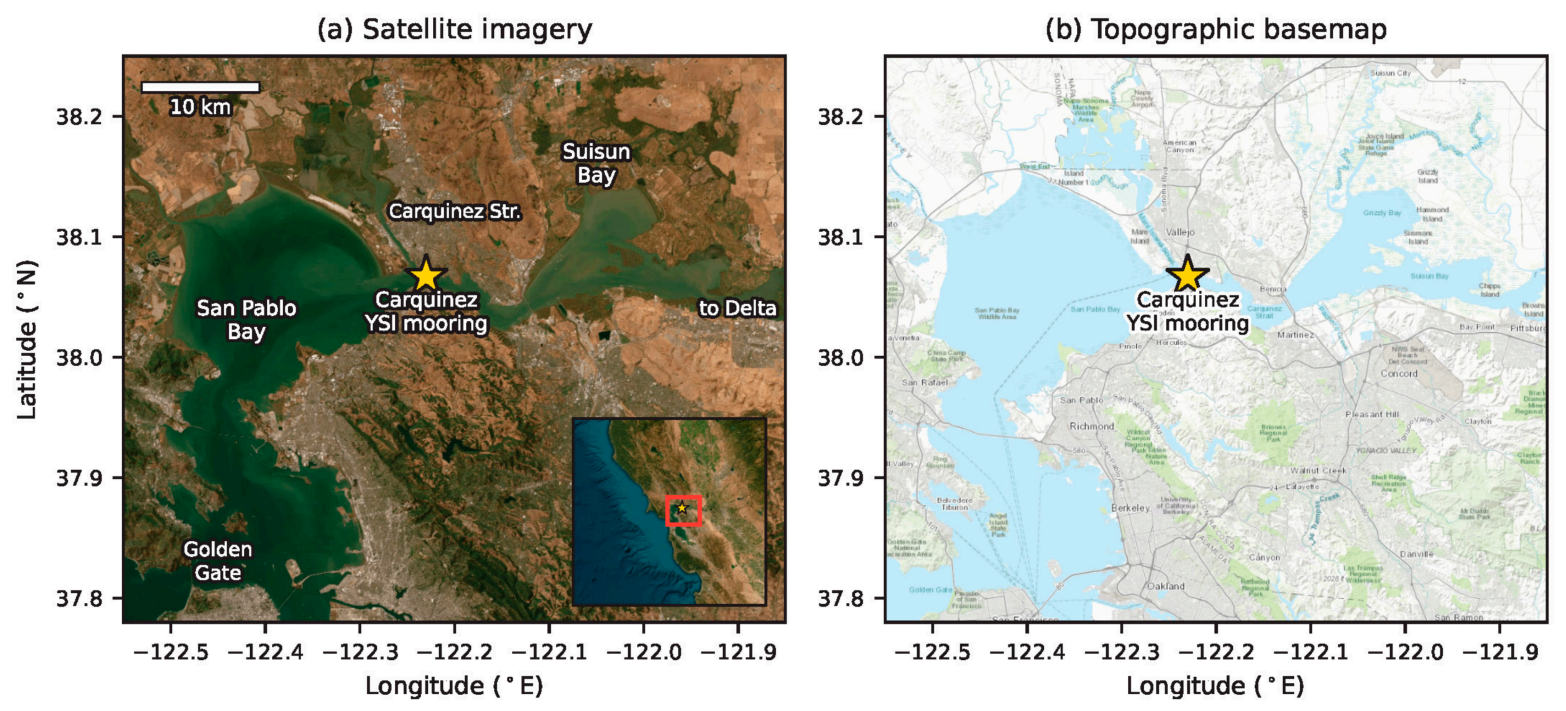
Study area. The Carquinez YSI mooring (star) at 38.067° N, 122.23° W, in Carquinez Strait between San Pablo Bay and Suisun Bay in the northern San Francisco Bay estuary. (**a**) Satellite imagery, with the main sub-embayments labeled and a California-context inset; (**b**) topographic basemap. Basemaps: Esri, Maxar, Earthstar Geographics.

The environmental and oceanic case is the forecasting of chlorophyll concentration at a fixed estuarine sensor mooring. The mooring sits in Carquinez Strait, in the northern reach of the San Francisco Bay estuary, at 38.067° N, 122.23° W, where the strait joins San Pablo Bay to Suisun Bay and the Sacramento–San Joaquin Delta (Figure 5). Chlorophyll at this location is set by several coupled, time-varying drivers. Freshwater inflow from the Delta, which differs strongly between the dry and the wet years of the California hydrological cycle, controls the salinity, the residence time and the nutrient supply of the water passing the strait; tides and wind resuspend sediment, so the suspended-particle load, and the light it leaves for phytoplankton, change from sub-daily to seasonal scales; and nutrient form and grazing further constrain the phytoplankton biomass that the chlorophyll signal measures [33,34,35]. Because these drivers act together and change between years, the chlorophyll series is non-stationary and its marginal distribution is not the same in different parts of the record.

**Figure 6:**
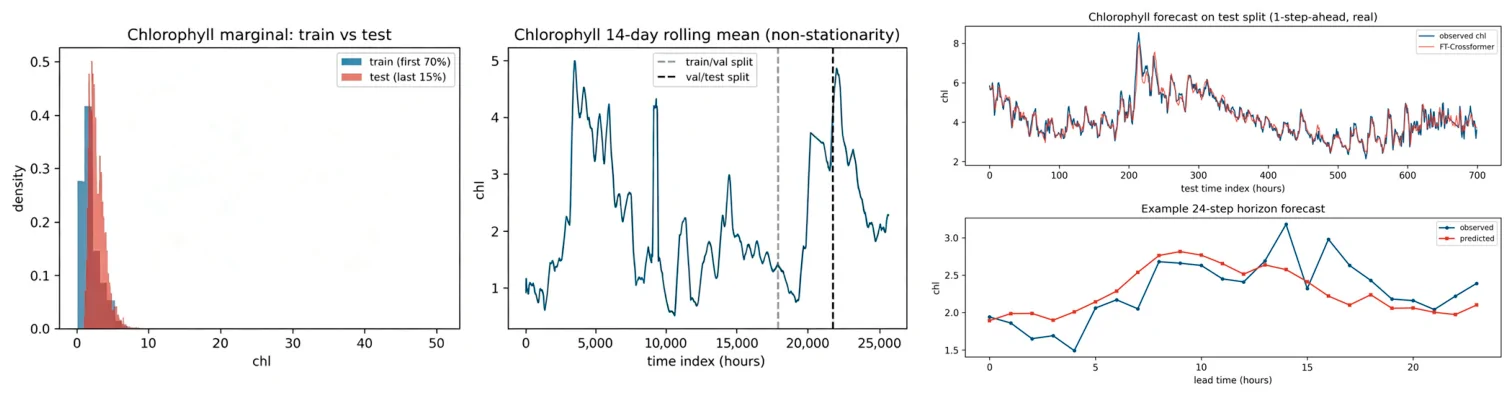
Real estuary chlorophyll from the Carquinez YSI mooring (ERDDAP rtcctdCMAysi). (**Left**): the train and test marginal distributions of chlorophyll, which differ (mean 1.99 against 2.58 μg/L, KS statistic 0.287). (**Right**): the FT-Crossformer prediction against the observed chlorophyll on the test segment at horizon τ=24.

The hourly record we use runs from September 2016 to August 2019, which spans the end of the 2012–2016 California drought and the wetter years that followed, so the freshwater forcing, and with it the chlorophyll regime, differs between the training and the test segments. The co-measured temperature, salinity, turbidity, dissolved oxygen and pH are observable proxies for these drivers, and the model forecasts chlorophyll from them while their joint distribution shifts; this is the setting the present method is built for. The record is served by NOAA CoastWatch ERDDAP (dataset rtcctdCMAysi from the San Francisco State University Estuary and Ocean Science Center) and contains 25,560 samples. The target variable is chlorophyll (chl, in μg/L), and the model also receives temperature, salinity, turbidity, dissolved oxygen and pH from the same mooring. We split the record chronologically, so the training and the test segment are disjointed in time. The chlorophyll mean rises from 1.99 to 2.58 μg/L across this split, and a two-sample Kolmogorov–Smirnov test on the marginal chlorophyll distribution gives a statistic of 0.287. The sensor stream therefore carries a clear distribution shift, which Figure 6 shows together with the prediction of the model against the observed chlorophyll.

Overall, the FT-Crossformer model can effectively simulate the changes in chlorophyll concentration as shown in the upper panel of Figure 6. Especially in the prediction of chlorophyll concentration of the nearly one-month period, the model can reflect the fluctuations in chlorophyll concentration with the spring and neap tide. Furthermore, the predicted series can also track the observed value closely, reproducing the rising and falling trend within 24 h, which is caused by tidal oscillation [36,37]. However, as shown in the lower panel of Figure 6, some short-term peak values may be smoothed out, which should be related to the abrupt hydrodynamic disturbances, or calm winds with high air temperature [38].

At the short, operationally relevant horizon τ=24, that is, next-day nowcasting from the hourly sensors, the full FT-Crossformer is the best of the four models we compare. Table 3 reports the mean-squared error and the mean absolute error on the real data, averaged over two seeds. The full model attains an MSE of 0.1848, below the 0.1873 of the variant without the frequency-domain weighting, the 0.1925 of the variant without the time-domain transformation and the 0.1912 of the Crossformer backbone. Removing the frequency-domain weighting raises the error, so this component contributes at the short horizon.

At this horizon, the ablation in Table 3 confirms that both proposed components contribute. Removing the frequency-domain weighting raises the MSE from 0.1848 to 0.1873, and removing the time-domain transformation raises it to 0.1925; the full model is below both, and it also improves on the Crossformer backbone, which attains 0.1912.

In the aforementioned time-series forecasting experiment, preprocessed multivariate environmental data are fed into the distribution transformation module of the FT-Crossformer, which temporarily detaches non-stationary statistical information from the data. Following transformation to the frequency domain via the Discrete Fourier Transform (DFT), the frequency-stability measurement module identifies task-relevant stable frequency components that are subsequently enhanced through adaptive weighting while unstable ones are suppressed. Upon restoration to the time domain via inverse DFT, the dimension-segmented embedding module preserves data contextualization and feeds into a multi-layer encoder–decoder architecture wherein the encoder, leveraging a two-stage attention mechanism, concurrently captures temporal dependencies encompassing seasonal, periodic, and short-term patterns alongside inter-variable correlations across the feature dimension, while the decoder generates layer-wise predictions that are subsequently recovered through the inverse transformation module to yield the final environmental variable forecasts. Thus, through the synergistic interplay of its constituent modules, FT-Crossformer enables accurate chlorophyll forecasting from authentic, distribution-shifted estuarine sensor streams, thereby furnishing an efficacious technical paradigm for intricate environmental surveillance.

### 4.4. Wastewater Nitrogen-And-Phosphorus Removal

**Figure 7:**
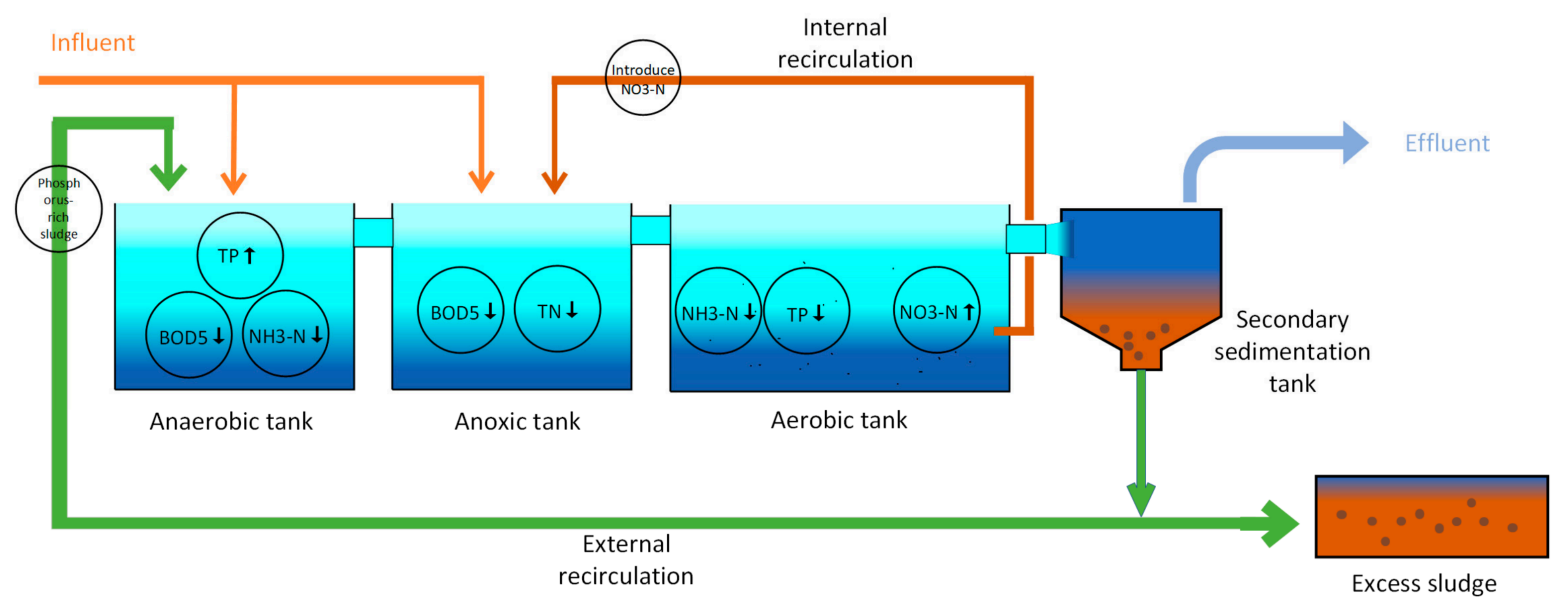
The anaerobic–anoxic–oxic (AAO) nitrogen-and-phosphorus-removal process. Online sensors on the influent and effluent streams provide the multivariate observations used for forecasting.

This case uses real data from a wastewater-treatment plant that runs the common anaerobic–anoxic–oxic (AAO) process for nitrogen and phosphorus removal, a biological process for nitrogen removal, phosphorus removal and the degradation of organic matter. The influent and the phosphorus-rich sludge that are returned from the secondary clarifier enter the anaerobic tank, where phosphorus-accumulating organisms release phosphorus and the absorption of organic matter lowers the biochemical oxygen demand and the ammonia concentration. In the anoxic tank the internal recycling yields nitrate and nitrite, and denitrifying bacteria reduce the organic matter to nitrogen gas. In the oxic tank, labeled the aerobic tank in Figure 7, nitrification continues, the ammonia concentration falls and the nitrate concentration rises while the phosphorus-accumulating organisms take up phosphorus in excess, and the secondary clarifier discharges the surplus sludge. Figure 7 shows the process, together with the online sensors that supply the forecasting inputs.

The data records 12 process variables from this plant between 25 September 2024 and 21 February 2025: influent chemical oxygen demand, influent ammonia, influent total phosphorus, influent total nitrogen, influent pH, influent flow, influent water temperature, nitrate concentration, effluent chemical oxygen demand, effluent ammonia, effluent total phosphorus and effluent total nitrogen. The sampling interval is ten minutes, which yields 18,986 samples. The training set and the test set are split by time, and their per-variable means and variances differ, so the operating data exhibits distribution shift over the recording period. These 12 variables are exactly the high-resolution sensor observations that this Special Issue is concerned with, and the prediction of the effluent nitrogen and phosphorus from the influent and operating sensors is an instance of an observation-to-state mapping.

We use the same setting as before. Table 4 reports the comparison over the prediction lengths. FT-Crossformer obtains the best result on every prediction length: relative to the original Crossformer, the average MSE drops by 63.9%, and relative to the second-best iTransformer it drops by 10.1%, which provides evidence for the accurate performance and the generalization capability of the method on real environmental sensor data.

### 4.5. Relation to Mechanistic Models and Data Assimilation

The two environmental cases support the reading of FT-Crossformer as a data-driven surrogate and a learned observation operator rather than as a generic forecaster. A mechanistic model of the AAO process, such as an activated sludge model [1], requires the repeated numerical solution of a stiff system of ordinary differential equations for each forward prediction, and its rate parameters must be calibrated to the plant. FT-Crossformer replaces that iterative stiff-ODE solve with a single forward pass of a trained network, paid once per window at deployment, while the training cost is paid once, offline. The model therefore addresses the first obstacle stated in the introduction, the computational cost of repeated forward prediction, by replacing the repeated numerical solution with a fixed-cost forward pass. We do not report a measured latency or FLOP comparison here, so this is a structural statement about the form of the computation rather than a measured speedup.

The model also addresses the second obstacle, the mapping from observations to states. Its input is the set of measured sensor variables and its output is the predicted process state, including effluent nitrogen and phosphorus, which are not measured at the same resolution; and the distribution-transformation and inverse-mapping pair is a learned quality-control and bias-correction step in the non-stationary sensor stream. In the language of data assimilation, FT-Crossformer can serve either as a fast surrogate for the forward model or as a learned observation operator inside an assimilation loop [27,39]. The model is a prediction model and not a variational or ensemble assimilation scheme; coupling it with a variational or ensemble method, so that the network supplies the forward operator or the observation operator and the assimilation supplies the uncertainty quantification, is a direction for future work.

## 5. Conclusions

We presented FT-Crossformer, a time–frequency joint-modeling method built on the Crossformer backbone that addresses the distribution shift and the variable coupling of non-stationary environmental sensor streams. The time-domain transformation, introduced via an affine transformation, mitigates distribution shift while preserving non-stationary information; concurrently, the frequency-domain weighting, implemented through stability quantification and adaptive weighting, amplifies the task-relevant frequency components that the transformation would otherwise leave unseparated. The public benchmarks and the ablation provide evidence supporting the generality and the contribution of each component, and the wastewater nitrogen-and-phosphorus-removal case shows accurate and robust prediction on real environmental sensor data. On real estuary chlorophyll data, the full model demonstrates superior accuracy at the next-day nowcasting horizon, and both proposed components contribute to this accuracy. The remaining limitations are the size and the computational cost of the model under resource-constrained deployment, which calls for a lighter model that meets low-latency requirements, and the coupling of the surrogate with a variational or ensemble assimilation scheme.

## Figures and Tables

**Table 1:** Public-benchmark comparison across prediction lengths. The best results are shown in bold. Each entry is the mean of three runs.

Data	*τ*	Ours		Crossformer		iTransformer		DLinear		FEDformer		Autoformer		Informer	
		MSE	MAE	MSE	MAE	MSE	MAE	MSE	MAE	MSE	MAE	MSE	MAE	MSE	MAE
ETTh1	24	**0.294**	**0.341**	0.306	0.367	0.304	0.359	0.312	0.355	0.318	0.384	0.439	0.440	0.577	0.549
	48	**0.337**	**0.365**	0.352	0.384	0.346	0.379	0.352	0.383	0.344	0.396	0.429	0.442	0.685	0.625
	168	**0.404**	**0.421**	0.434	0.443	0.428	0.429	0.416	0.430	0.412	0.449	0.493	0.479	0.931	0.725
	336	**0.456**	**0.442**	0.581	0.555	0.490	0.462	0.481	0.452	0.459	0.474	0.509	0.492	1.128	0.873
	720	**0.487**	**0.468**	0.608	0.575	0.519	0.493	0.509	0.516	0.522	0.515	0.539	0.537	1.241	0.896
ETTm1	24	**0.192**	**0.268**	0.231	0.293	0.223	0.296	0.217	0.289	0.290	0.364	0.410	0.428	0.323	0.369
	48	**0.271**	**0.319**	0.307	0.353	0.309	0.355	0.281	0.330	0.342	0.396	0.485	0.464	0.494	0.503
	96	**0.311**	**0.348**	0.332	0.384	0.341	0.376	0.345	0.371	0.366	0.412	0.502	0.476	0.678	0.614
	288	**0.393**	**0.404**	0.482	0.495	0.406	0.414	0.401	0.418	0.422	0.433	0.604	0.522	1.056	0.786
	672	**0.455**	**0.443**	0.549	0.518	0.487	0.458	0.489	0.462	0.457	0.464	0.617	0.530	1.192	0.926
ETTh2	96	**0.177**	**0.287**	0.259	0.375	0.297	0.349	0.295	0.352	0.358	0.397	0.346	0.388	3.755	1.525
	192	**0.216**	**0.315**	0.274	0.368	0.380	0.398	0.452	0.462	0.429	0.439	0.456	0.452	5.602	1.931
	336	**0.263**	**0.360**	0.301	0.419	0.423	0.432	0.504	0.490	0.496	0.487	0.482	0.486	4.721	1.835
	720	**0.301**	**0.382**	0.489	0.509	0.430	0.447	0.577	0.538	0.463	0.474	0.515	0.511	3.647	1.625
ETTm2	96	**0.119**	**0.233**	0.131	0.241	0.180	0.264	0.183	0.273	0.203	0.287	0.255	0.339	0.365	0.453
	192	**0.157**	**0.264**	0.169	0.284	0.250	0.309	0.260	0.325	0.269	0.328	0.281	0.340	0.533	0.563
	336	**0.185**	**0.290**	0.242	0.338	0.311	0.348	0.336	0.413	0.325	0.366	0.339	0.372	1.363	0.887
	720	**0.243**	**0.336**	0.433	0.417	0.412	0.407	0.415	0.453	0.421	0.415	0.433	0.432	3.379	1.338
ILI	24	**2.002**	**0.863**	3.702	1.263	2.332	1.036	2.940	1.205	2.687	1.147	3.101	1.238	4.588	1.462
	36	**2.075**	**0.887**	3.732	1.251	2.238	1.015	2.826	1.184	2.887	1.160	3.397	1.270	4.845	1.496
	48	**2.089**	**0.897**	4.166	1.333	2.213	1.013	2.677	1.155	2.797	1.155	2.947	1.203	4.865	1.516
	60	**2.117**	**0.927**	4.664	1.426	2.251	1.034	3.011	1.245	2.809	1.163	3.019	1.202	5.212	1.576
Traffic	24	**0.344**	**0.240**	0.491	0.281	0.350	0.249	0.351	0.261	0.562	0.375	0.550	0.363	0.608	0.334
	48	**0.394**	**0.251**	0.519	0.295	0.399	0.259	0.410	0.272	0.567	0.378	0.595	0.407	0.644	0.359
	168	0.431	**0.269**	0.513	0.289	**0.417**	0.278	0.423	0.287	0.607	0.389	0.649	0.388	0.660	0.391
	336	0.462	**0.279**	0.530	0.300	**0.429**	0.284	0.436	0.296	0.624	0.385	0.624	0.405	0.747	0.405
Weather	96	**0.151**	**0.201**	0.158	0.237	0.174	0.220	0.186	0.245	0.217	0.296	0.266	0.336	0.313	0.402
	192	**0.202**	**0.249**	0.212	0.284	0.221	0.256	0.221	0.282	0.276	0.336	0.307	0.367	0.367	0.430
	336	**0.258**	**0.289**	0.273	0.347	0.281	0.298	0.265	0.319	0.339	0.380	0.359	0.395	0.398	0.428
	720	**0.342**	**0.344**	0.381	0.407	0.358	0.353	0.353	0.362	0.403	0.428	0.419	0.428	0.712	0.610
Exchange	96	**0.079**	**0.204**	0.231	0.363	0.089	0.216	0.088	0.218	0.148	0.278	0.197	0.323	0.847	0.752
	192	**0.174**	**0.301**	0.525	0.551	0.186	0.312	0.176	0.315	0.271	0.315	0.301	0.369	1.204	0.895
	336	**0.311**	**0.413**	0.994	0.806	0.337	0.431	0.313	0.427	0.460	0.429	0.509	0.524	1.672	1.036
	720	**0.857**	**0.690**	1.151	0.999	0.898	0.699	0.869	0.695	1.195	0.693	1.447	0.941	2.478	1.310

**Table 2:** Ablation on ETTh2 and ILI. Best results are shown in bold.

Data	*τ*	Ours		w/o FDW		w/o DTIM		w/o DSETA	
		MSE	MAE	MSE	MAE	MSE	MAE	MSE	MAE
ETTh2	96	**0.177**	**0.287**	0.187	0.297	0.297	0.354	0.224	0.290
	192	**0.216**	**0.315**	0.228	0.325	0.454	0.464	0.360	0.368
	336	**0.263**	**0.360**	0.280	0.376	0.506	0.492	0.399	0.397
	720	**0.301**	**0.382**	0.324	0.403	0.587	0.548	0.446	0.432
ILI	24	**2.001**	**0.863**	2.034	0.887	2.677	1.157	2.287	1.047
	36	**2.075**	**0.887**	2.088	0.899	2.816	1.174	2.387	1.060
	48	**2.088**	**0.898**	2.153	0.919	2.930	1.195	2.497	1.115
	60	**2.115**	**0.925**	2.217	0.959	3.011	1.245	2.509	1.133

**Table 3:** Real estuary chlorophyll (Carquinez YSI mooring, ERDDAP rtcctdCMAysi) at horizon τ = 24, mean over two seeds. Best results are shown in bold.

Model	MSE	MAE
FT-Crossformer (full)	**0.1848**	**0.3002**
w/o FDW (RevIN only)	0.1873	0.3034
w/o DTIM (FDW only)	0.1925	0.3027
Crossformer (backbone)	0.1912	0.3050

**Table 4:** Wastewater AAO nitrogen-and-phosphorus-removal prediction across prediction lengths. Best in bold.

Data	*τ*	Ours		iTransformer		Crossformer		Autoformer		Informer	
		MSE	MAE	MSE	MAE	MSE	MAE	MSE	MAE	MSE	MAE
AAO	24	**0.190**	**0.248**	0.227	0.298	0.416	0.480	0.412	0.445	1.601	1.081
	48	**0.273**	**0.322**	0.313	0.347	0.647	0.619	0.471	0.492	2.034	1.214
	96	**0.314**	**0.358**	0.339	0.397	0.855	0.747	0.498	0.519	2.338	1.307
	168	**0.367**	**0.398**	0.392	0.415	1.423	0.993	0.551	0.538	2.512	1.338
	336	**0.491**	**0.462**	0.535	0.491	1.643	1.001	0.603	0.569	2.630	1.385

## Data Availability

The estuary chlorophyll data from the Carquinez YSI mooring (ERDDAP rtcctdCMAysi) used in this study are openly available in the San Francisco State University Estuary and Ocean Science Center (https://www.diver.orr.noaa.gov/ accessed on 3 February 2026), meanwhile the wastewater data comes from the private factory and cannot be made public.
